# Measles IgG Antibody Index Correlates with T2 Lesion Load on MRI in Patients with Early Multiple Sclerosis

**DOI:** 10.1371/journal.pone.0028094

**Published:** 2012-01-19

**Authors:** Berit Rosche, Sarah Laurent, Silja Conradi, Jörg Hofmann, Klemens Ruprecht, Lutz Harms

**Affiliations:** 1 Department of Neurology and Experimental Neurology, Charité – Universitätsmedizin Berlin, Berlin, Germany; 2 Department of Virology, Charité – Universitätsmedizin Berlin, Berlin, Germany; La Jolla Institute for Allergy and Immunology, United States of America

## Abstract

**Background:**

B cells and humoral immune responses play an important role in the pathogenesis and diagnosis of multiple sclerosis (MS). A characteristic finding in patients with MS is a polyspecific intrathecal B cell response against neurotropic viruses, specifically against measles virus, rubella virus, and varicella zoster virus, also known as an MRZ reaction (MRZR). Here, we correlated from the routine clinical diagnostics individual IgG antibody indices (AIs) of MRZR with magnetic resonance imaging (MRI) findings in patients with first MS diagnosis.

**Methods/Results:**

MRZR was determined in 68 patients with a clinically isolated syndrome (CIS) or early relapsing-remitting MS (RRMS). Absolute AI values for measles virus, rubella virus, and varicella zoster virus were correlated with T2 lesion load and gadolinium enhancing lesions on cerebral MRI (cMRI) and cMRI combined with spinal MRI (sMRI). Measles virus AI correlated significantly with T2 lesion load on cMRI (p = 0.0312, Mann-Whitney *U* test) and the sum of lesions on cMRI and sMRI (p = 0.0413). Varicella zoster virus AI also showed a correlation with T2 lesion load on cMRI but did not reach statistical significance (p = 0.2893).

**Conclusion:**

The results confirm MRZR as part of the polyspecific immune reaction in MS with possible prognostic impact on MRI and clinical parameters.

Furthermore, the data indicate that intrathecal measles virus IgG production correlates with disease activity on cMRI and sMRI in patients with early MS.

## Introduction

Great effort has done into defining prognostic markers in multiple sclerosis (MS) over recent years. To date, a combination of clinical, cerebrospinal fluid (CSF) and MRI variables are used to predict disease course. Research has shown high T2 lesion load on cMRI at diagnosis is associated with a more active disease course [1], [2] and in clinical routine, T2 lesion load and gadolinium enhancement are the most important markers in evaluating disease activity.

CSF is a promising source of biochemical markers in MS, as its compounds are relevant not only in the diagnosis of MS but may also reflect disease activity. Detection of oligoclonal bands in CSF and at least two positive AI against measles virus, rubella virus and varicella zoster virus of ≥1.5 (MRZR), are important markers in the diagnosis of MS. In 80–100% of patients with MS, a polyspecific intrathecal B cell response in the form of a positive MRZR is detectable in CSF and is accepted as being highly specific for this disease [3], [4]. Furthermore, other studies have shown a correlation between disease activity and both intrathecal IgM synthesis and an elevated B cell monocyte ratio, which emphasizes the importance of humoral response in MS [5]–[7].

In the present study, we correlated from the routine clinical diagnostics absolute AIs for measles virus, rubella virus, and varicella zoster virus (VZV) with magnetic resonance imaging (MRI) parameters of disease activity in patients with a first diagnosis of clinically isolated syndrome (CIS) or early RRMS.

## Methods

### Patients

61patients with relapsing-remitting MS according to the revised McDonald criteria from 2010 [8] and 7 patients with a CIS, who were treated at the Department of Neurology, Charité – Universitätsmedizin Berlin between 2007 and 2010, were enrolled in this study. All patients presented with first clinical symptoms of the disease and underwent lumbar puncture and cerebral MRI as part of the routine diagnostic work-up. Additionally, 44 patients in the group underwent spinal MRI. All patients had positive oligoclonal bands in their CSF. At the time of investigation no patient was being treated with steroids or immunomodulatory or immunosuppressive substances. The patient data was anonymized for the analysis and the study was approved by Charité University Hospital ethics committee. We do not have informed consent from our patients. According to Charité University Hospital ethics committee in case of use of routine parameters and anonymized data it is not necessary to obtain informed consent from patients.

### Determining MRZ reaction

Antibodies against measles virus and VZV were measured in serum and CSF samples using commercial enzyme-linked immunosorbent assays (ELISA; Enzygnost, Siemens Healthcare Diagnostics, Germany) according to the manufacturer's instructions. Antibodies against rubella virus were also measured by ELISA (medac, Wedel, Germany). A standard curve consisting of serial dilutions of a standard human plasma (SHP, Siemens Healthcare Diagnostics) was included on each ELISA plate. The same batch of SHP was used for all experiments. Total albumin and IgG concentration in serum (IgG (serum)) and CSF were measured nephelometrically (BN ProSpec, Siemens Healthcare Diagnostics).

AI values were calculated either with the formula: AI = (IgG_spec_ CSF/IgG_spec_ serum)/(IgG_total_ CSF/IgG_total_ serum) = Q_spec_/Q_IgG_ if Q_IgG_<Q_Lim_, or AI = Q_spec_/Q_Lim_ if Q_IgG_>Q_Lim_. Q_Lim_ was calculated as described previously (Reiber and Peter, 2001). AI values ≥1.5 were considered as indicative of an intrathecal virus-specific antibody production.

### MRI analysis

All 67 patients underwent cerebral MRI concurrently with the spinal tap. The majority of patients were scanned on a 1.5 T MRI unit according to a a previously fixed protocol including T1-weighted spin-echo (SE) axial slices with and without application of gadolinium as well as T2-weighted SE axial slices. Hyperintense lesion on T2-weighted MRI lesions >3 mm^2^ were analyzed and quantified on hardcopies. For this study, the presence of ≥1 lesions suggestive of MS in T2 weighted MRI as well as Barkhof criteria [1] were used as diagnostic criteria.

*Statistical analysis:* Statistical significance of differences in absolute AI values between patients with high and low lesion loads in T2-weighted images and patients with and without Gd-enhancing lesions were analysed by Mann-Whitney *U* test using GraphPad Prism 5 software. P values <0.05 were considered significant.

## Results

**Figure 1:**
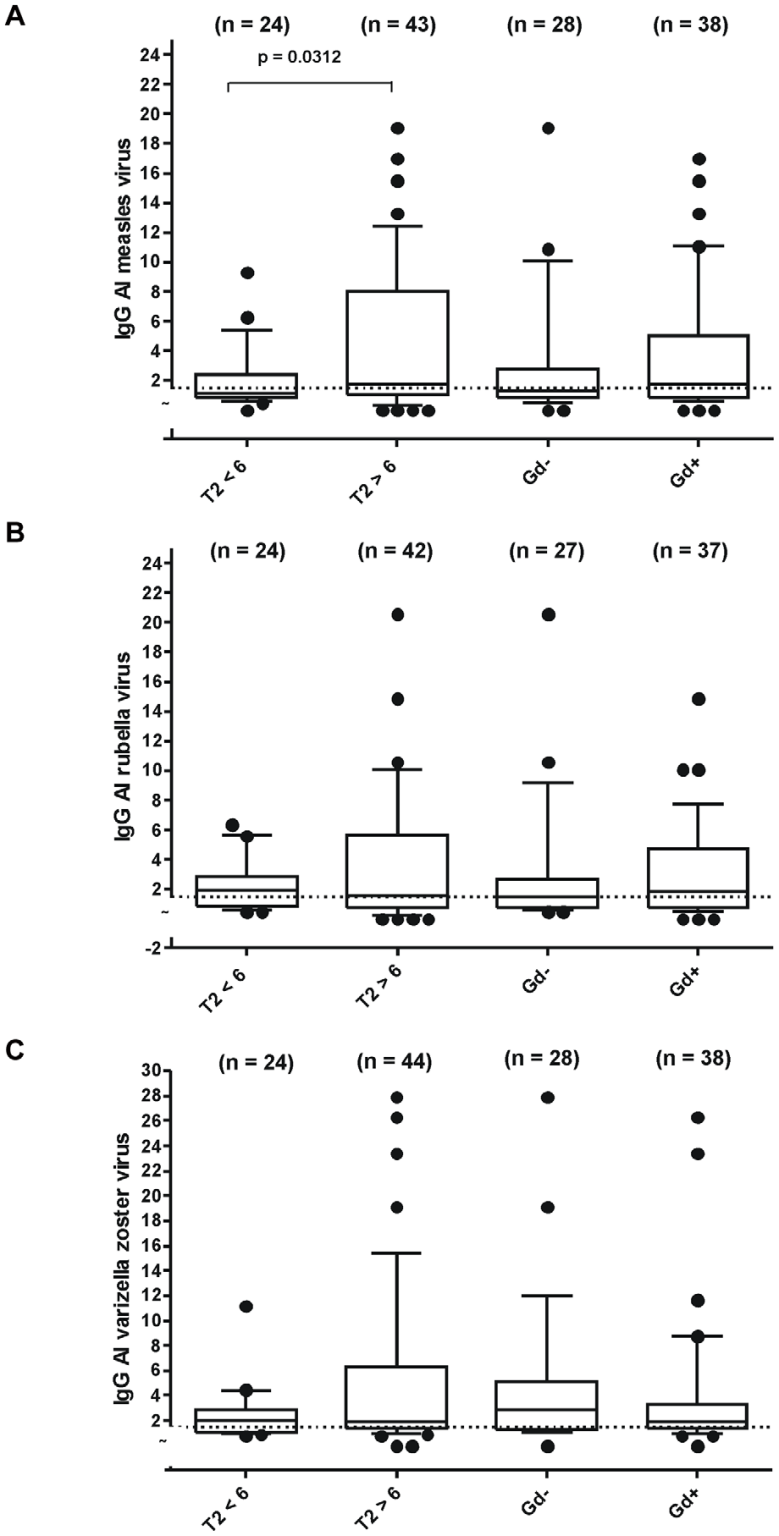
IgG antibody index of MRZR and MRI activity. IgG antibody index (AI) results for measles virus (A), rubella virus (B) and varicella zoster virus (C) for patients with <6 lesions, ≥6 lesions, no Gd-enhancing lesions and Gd-enhancing lesions on cMRI are shown as box plot graphs. The boxes include values between the 25^th^ and 75^th^ percentile of the distribution, while the line within the box represents the median value. The whiskers above and below the box indicate the 90^th^ and 10^th^ percentile, respectively, and the black dots indicate the outliers, while the dotted line at AI 1.5 indicates the upper limit of the reference range. The number of patients in each group is shown in brackets above boxes.

The demographic data of all patients included in this study are shown in Table 1. A positive MRZ reaction as defined by a combination of at least two positive AIs was present in 38 of 66 patients (57.57%). The German Multiple Sclerosis Therapy Consensus Group supposes an increased MS risk in CIS patients with ≥6 T2 lesions and recommends an early therapy for these patients because of higher disease activity [9]. To correlate AI levels with T2 lesion load on cMRT, the patients were divided into two groups: those with <6 lesions and those with ≥6 lesions. Mean absolute AI values for measles virus, rubella virus, and varicella zoster virus were compared between both groups. Patients with ≥6 T2 lesions had a higher mean measles AI than patients with <6 T2 lesions (p = 0.0312). For 44 patients, both cerebral and spinal MRI was available. When cerebral and spinal T2 lesions were taken together, a significant difference of mean measles virus AI values between patients with higher (≥6) and lower (<6) lesion load was observed (p = 0.0413, see Figure 1A). The comparison of AI means against VZV revealed a tendency toward a higher AI in the group of patients with higher lesion load, which did not reach statistical significance (p = 0.2893, see Figure 1C). For rubella virus, mean absolute AI values did not differ between the two groups (see Figure 1B).

**Table 1:** Demographic data of all 68 patients. MRZR+ = AI for measles virus, rubella virus, varicella zoster virus, two or more AI≥1.5.

	n Patients	Patients in %
**(Female/male)**	48/20	71%/29%
**Age (years) at MS onset as median (range)**	30 (18–64)	
**Sample acquisition age (years) as median (range)**	31 (18–64)	
**Diagnosis CIS/RRMS**	7/61	10,29%/89,71%
**Measles AI≥1.5/<1.5**	31/36	46.27%/53.73%
**Rubella AI≥1.5/<1.5**	36/30	54.54%/45.46
**Zoster AI≥1.5/<1.5**	47/21	69.12%/30.88
**MRZR+**	38	57.57%

But rubella virus AI also showed a correlation with Gd-enhancing lesions on combined cerebral and spinal MRI but did not reach statistical significance (p = 0.0611). Furthermore, none of the other AIs correlated with the presence of Gd-enhancing lesions (see Figure 1).

We also performed a Spearman Rank Correlation for single AIs and the number of T2 or Gd-enhancing lesions, which did not yield any statistically significant results (see Figure S1).

## Discussion

We investigated the relationship between the levels of the individual IgG AIs that form the MRZ reaction and quantitative MRI outcomes in patients with MS. Higher anti-measles AI levels were associated with a higher T2 lesion load in cMRI and sum of T2 lesions in cerebral and spinal MRI. There was no link between individual MRZ-IgG AIs and Gd-enhancing lesions as a marker of acute inflammation on cMRI.

MRZ reaction in CSF is positive in chronic CNS autoimmune diseases (e.g. neurolupus or CNS-vasculitis), but has been proposed as a specific marker for autoimmunity in MS [4]. Positive detection varies at between 42% and >90% in newly diagnosed CIS or MS patients depending on the inclusion criteria used by the study in question [10]. We detected a positive MRZ reaction in 57.57% of our patients, with VZV as the most frequent single AI. Brettschneider et al. defined a scoring system based on the MRZR to predict the development of relapsing-remitting MS (RRMS) from CIS, and found these AI indicators to have more prognostic value than the detection of oligoclonal bands and MRI parameters [11]. To date MRZR has been understood as polyspecific CNS antibody production directed against diverse common neurotropic viruses [12]–[14]. Intrathecal IgG synthesis in MS is widely known to be stable and clonally uniform over time [15] and possibly high MRZ AIs signal indicate B cell activity, which has been associated with more severe disease progression in previous studies [5]. In our study we categorized the patients into two groups, namely those with low initial lesion numbers (less than 6) and those with a high number of lesions (6 or more) and see significant higher measles AI levels in the last group but failed to show a direct correlation between viral IgG AI levels and T2 lesion load in MRI (see Figure S1). We explain this finding with the above mentioned observation that oligoclonal bands and intrathecal IgG production are stable markers [15] that may reflect overall disease activity. In MS cMRI lesion load usually increases over time while IgG production is stable (see Figure S2). As a consequence we can not expect a direct correlation between both parameters. For this reason both markers (IgG level and IgG AI) correlate with number of MRI lesions only at the beginning of the disease and may have prognostic implication (see Figure S3).

On the other hand MRI T2 lesion load has an important clinical impact in diagnosis of MS and therapeutic decisions. The number of lesions in CIS correlates with the risk of conversion to clinically definite MS and is used to monitor disease activity [16].

In conclusion, our data suggest that measles AIs correlate with MRI markers in early MS and increased measles AI levels are a marker of increased disease activity. The study underlines the relevance of humoral immunity in MS pathogenesis and supports MRZR as an important tool not only in MS diagnosis but also to estimate disease activity. Additional longitudinal studies are needed to investigate the relationship between MRZ AIs and MRI parameters on one hand and clinical aspects like neurocognitive function and disability progression on the other.

## Supporting Information

Figure S1 **Measle virus IgG AI and T2 lesion load in MRI.** Correlation between number of T2 lesions in cerebral MRI (A), including 67 patients, and sum of lesions in cerebral and spinal MRI (sMRI) (B) from 43 patients with IgG AI for measles virus.
(TIF)

Figure S2 **Correlation between symptom duration and total intrathecal IgG synthesis.** Correlation of time between first symptoms (FS) and lumbal puncture (LP) and total intrathecal IgG synthesis from 65 patients.
(TIF)

Figure S3 **Total intrathecal IgG and T2 and Gd-enhancing lesion load in MRI.** Comparison of mean total intrathecal IgG for patients with <6 lesions and ≥6 lesions in cMRI (A) and both cMRI and sMRT (B). Comparison of mean total intrathecal IgG for patients with Gd-enhancing and no Gd-enhancing lesions in cMRI (C) and both cMRI and sMRI (D).
(TIF)
