# Supplementary figures and images for: Measles IgG Antibody Index Correlates with T2 Lesion Load on MRI in Patients with Early Multiple Sclerosis
Source: PLoS One. 2012 Jan 19;7(1):e28094. doi: 10.1371/journal.pone.0028094 (PMC3261854; doi:10.1371/journal.pone.0028094)

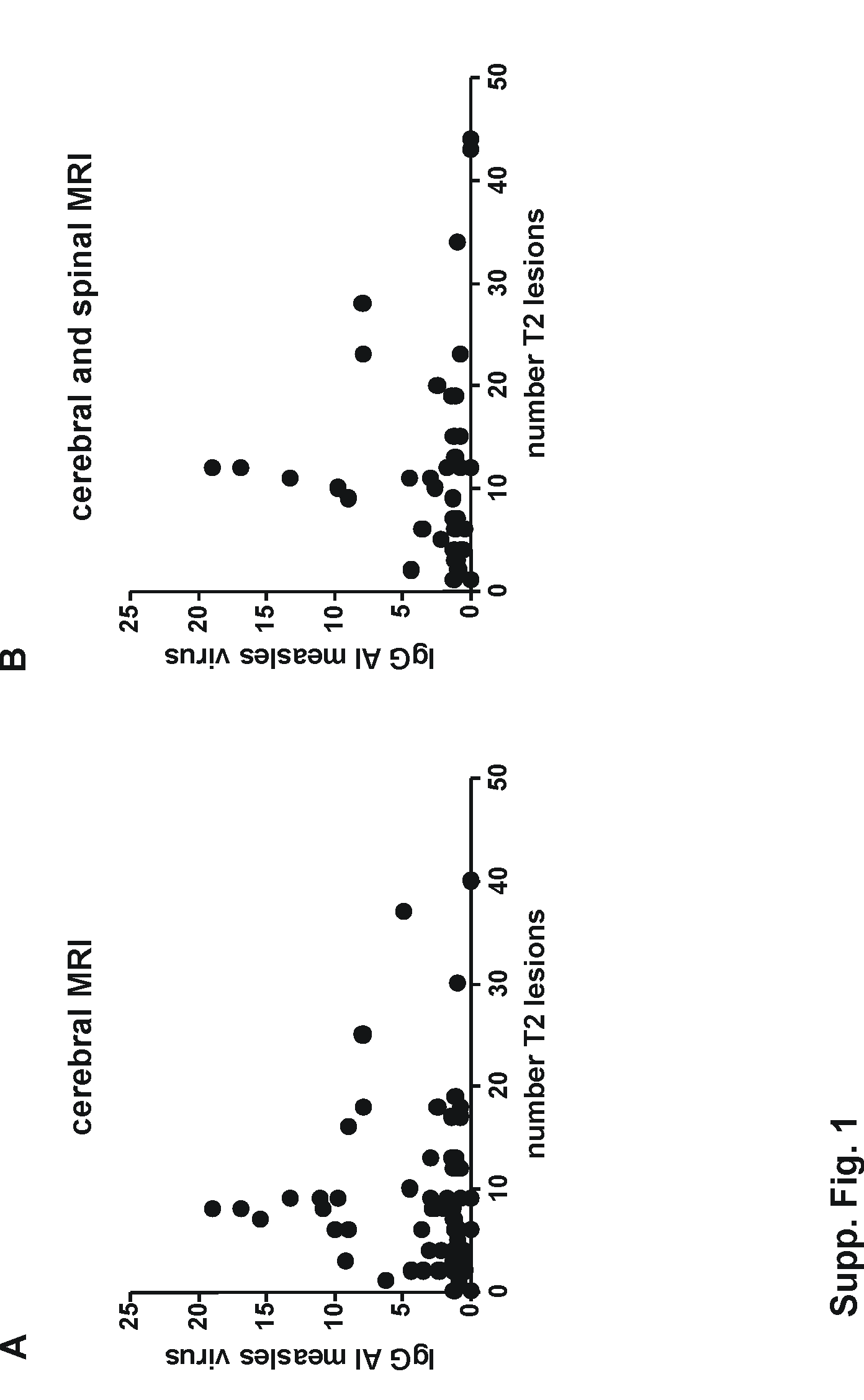

Supplement: Figure S1 — Measle virus IgG AI and T2 lesion load in MRI. Correlation between number of T2 lesions in cerebral MRI (A), including 67 patients, and sum of lesions in cerebral and spinal MRI (sMRI) (B) from 43 patients with IgG AI for measles virus. (TIF) [file pone.0028094.s001.tif]

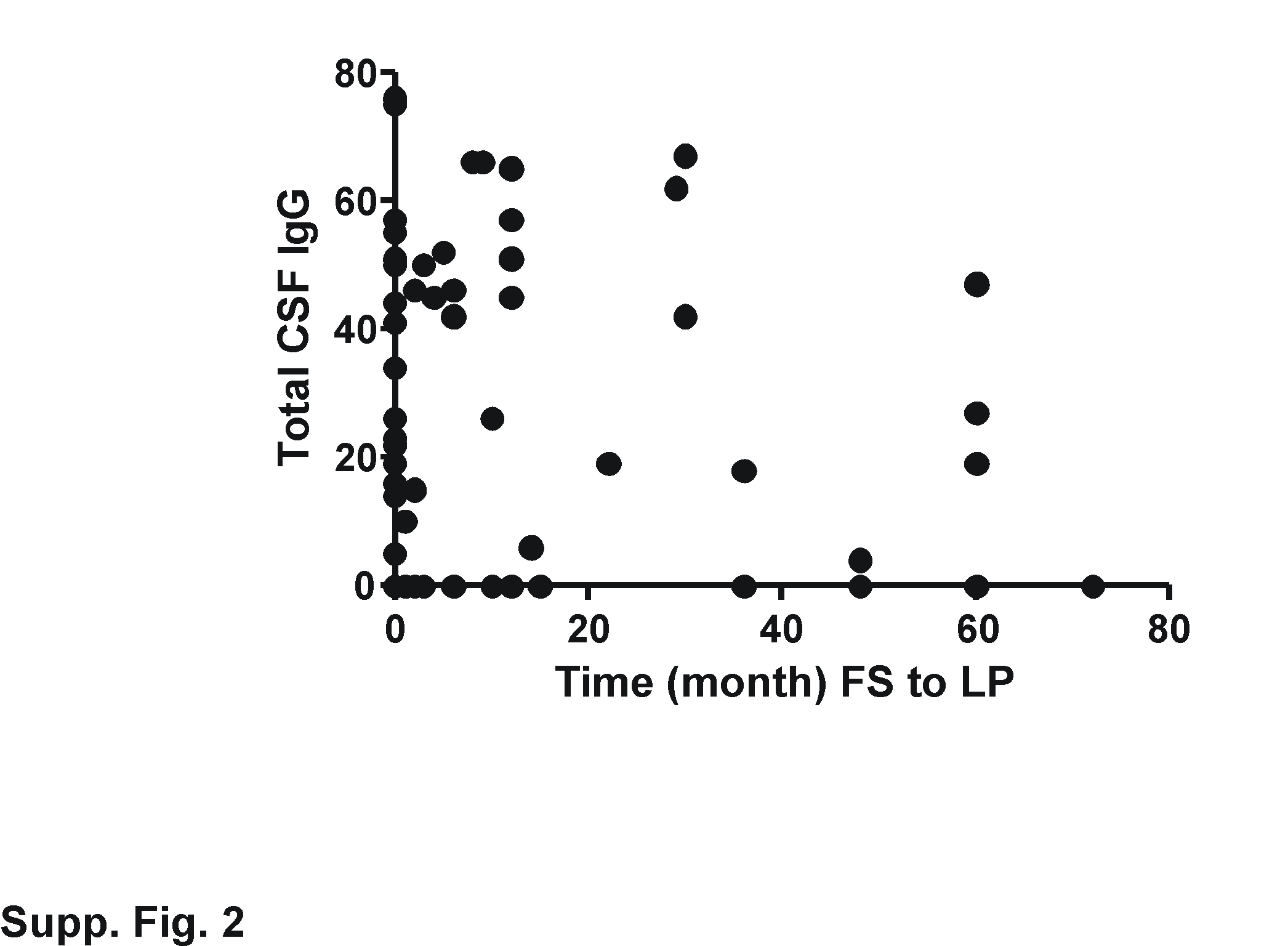

Supplement: Figure S2 — Correlation between symptom duration and total intrathecal IgG synthesis. Correlation of time between first symptoms (FS) and lumbal puncture (LP) and total intrathecal IgG synthesis from 65 patients. (TIF) [file pone.0028094.s002.tif]

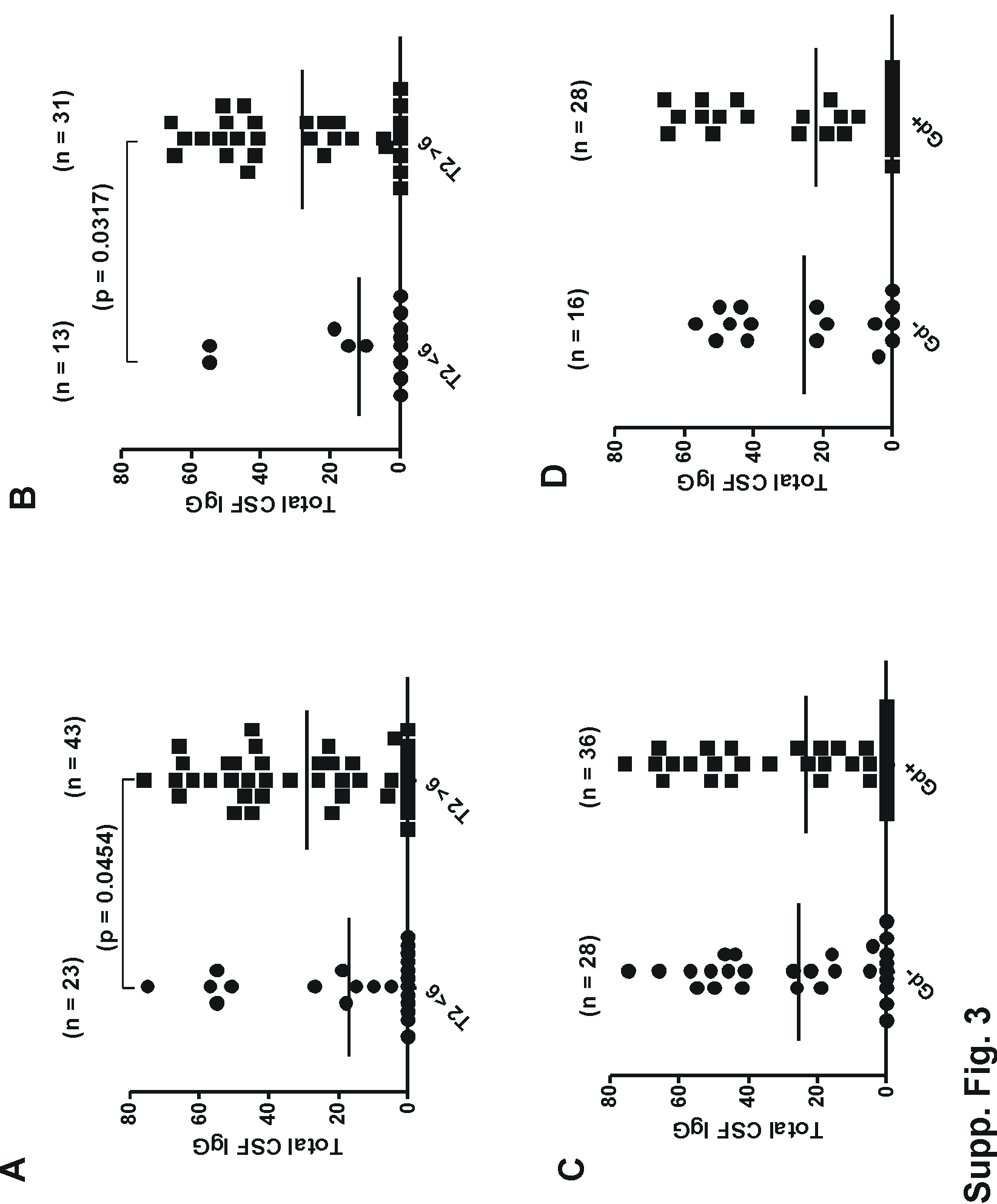

Supplement: Figure S3 — Total intrathecal IgG and T2 and Gd-enhancing lesion load in MRI. Comparison of mean total intrathecal IgG for patients with <6 lesions and ≥6 lesions in cMRI (A) and both cMRI and sMRT (B). Comparison of mean total intrathecal IgG for patients with Gd-enhancing and no Gd-enhancing lesions in cMRI (C) and both cMRI and sMRI (D). (TIF) [file pone.0028094.s003.tif]
